# Increased Neutrophil Respiratory Burst Predicts the Risk of Coronary Artery Lesion in Kawasaki Disease

**DOI:** 10.3389/fped.2020.00391

**Published:** 2020-07-28

**Authors:** Jing Hu, Wei Qian, Zhiwei Yu, Tao Xu, Liang Ju, Qi Hua, Yan Wang, Jing Jing Ling, Haitao Lv

**Affiliations:** ^1^Children's Hospital of Soochow University, Suzhou, China; ^2^Wuxi Children's Hospital Affiliated to Nanjing Medical University, Wuxi, China

**Keywords:** kawasaki disease, coronary artery lesion, neutrophil, flow cytometry, brain natriuretic peptide

## Abstract

**Background:** Kawasaki diseases (KD) is a febrile systemic vasculitis in infants associated with coronary aneurysm. The etiology of KD remains unclear. Human neutrophils have great capacity to cause tissue damage in inflammatory diseases via their inappropriate activation to release reactive oxygen species (ROS). Brain natriuretic peptide (BNP) is a substantial modulator of neutrophil activation to regulate ROS production. It is increasingly released from the myocardium in heart failure and myocardial inflammatory states.

**Objective:** The purpose of this study was to explore the potential role of neutrophil respiratory burst in the pathogenesis of coronary artery lesions (CAL) in KD.

**Materials and Methods:** A total of 78 children were enrolled. Of all the cases, 20 cases are healthy control (HC), 20 are with coronary artery lesion (CAL), and 38 are with non-coronary artery lesion (NCAL). The activation ratio of neutrophils was evaluated by flow cytometry. In addition, plasma levels of BNP were detected.

**Results:** Our results showed that the activation ratio of neutrophils in KD with CAL is significantly higher than the other two groups (HC and NCAL). Besides, the plasma levels of BNP in KD (with or without CAL) were higher than that in HC.

**Conclusions:** These findings suggested that neutrophil respiratory burst may play a significant role in the pathogenesis of CAL, and predicts the risk of CAL in Kawasaki disease.

## Introduction

Kawasaki disease (KD), first reported by the Japanese physician Tomisaku Kawasaki, is an acute febrile disease characterized as systemic vasculitis. Almost 50 years have passed since its initial description. The incidence of KD is increasing worldwide, and in more economically developed countries, KD is now the most common cause of acquired heart disease in children. The cause or etiology and pathogenesis of KD is still being debated. Previous pathological studies in KD patients showed all cardiac tissues are associated with the acute inflammatory phase of the disease. Vasculitis leads to the destruction of normal arterial structure, followed by aneurysm expansion, especially affecting the proximal coronary artery, destruction of intima and media, and replacement of myocytes by fibroblasts and connective tissue (1). The most important complication is serious coronary artery lesions (CAL). It may lead to serious formation of coronary aneurysm, which is a major cause of cardiac sequelae such as myocardial infarction (MI) and sudden death. These events are caused by coronary stenosis due to intimal proliferation or thrombotic formation (2). Timely high-dose intravenous immunoglobulin (IVIG) treatment effectively resolves the inflammation and reduces the occurrence of CAL in patients with KD (3–5). The reduction of risk for CAL by IVIG therapy in KD may be due partially to the decreased number of activated neutrophils in circulation (6).

Neutrophils are involved in the damage that occurs in coronary arteries in the early stage of KD, especially in the patient who died on the 10th day of the course of KD. Vasodilation might occur as a result of injury to vascular walls caused by neutrophil. Neutrophil infiltration reached a peak earlier than the infiltrations of monocytes/macrophages and lymphocytes. A large number of neutrophils were found to deform and penetrate the coronary artery wall in dead cases (7). Study revealed that the apoptosis of peripheral neutrophils was down-regulated persistently during the acute phase of KD (8). This suggests that the prolonged life span of activated neutrophils may contribute to the pathogenesis of KD vasculitis.

Neutrophils are important innate immune cells, which play a role of phagocytosis and killing in innate immunity and participate in infectious inflammation and other inflammatory reactions. When microbial pathogens, especially pyogenic bacteria, invade and inflammation occurs, neutrophils rapidly recruited and tended to inflammation sites mediated by various inflammation and cytokines, killing and eliminating pathogens by secreting cytokines, degranulation, and release reactive oxygen species (ROS) by the NADPH oxidase system, which is called respiratory burst. Neutrophil respiratory burst is related to the short but significant increase of neutrophil's absorption and utilization of oxygen, which is also characterized by the release of cytokines, the activation of monocytes and macrophages, and the increased release of ROS to extracellular space (9). But neutrophils inappropriate respiratory burst and elevated concentrations of ROS induce cell death and perpetuate more highly reactive radicals that lead to adverse cardiac remodeling might cause sustained tissue infiltration with neutrophils and monocytes, and persistent vasomotor dysfunction (10).

Brain natriuretic peptide (BNP) is mainly synthesized in ventricular myocytes and secreted by the left ventricle through the coronary sinus into the circulation. The production of BNP is regulated by the stretch of cardiac wall caused by myocardial volume overload, and BNP is increasingly released into systemic circulation in many clinical diseases characterized by heart failure and/or myocarditis (11). Recently a study found that under physiological conditions, BNP may play a potential regulatory role in neutrophil respiratory burst and ROS production (12). Thus, to investigate the role of neutrophils respiratory burst in KD, we detected the ROS production and the level of serum BNP in children with KD.

## Materials and Methods

### Study Design and Subjects

A total of 78 children were recruited from January to December in 2019. For the healthy control group, inpatients with inguinal hernia and other selected surgery eliminating fever, allergic purpura, and other immune diseases were selected. We asked the parents to provide an additional blood sample (2 ml from their children). Patients who have a course of disease more than 10 days or have been treated with IVIG were not included. All of the KD patients were identified according to the criteria proposed by the Japanese Circulation Society Joint Working Group. Echocardiography was performed at 1, 2, and 4 weeks after the onset of fever. CALs were diagnosed on the basis of the Z scores of the left main coronary artery, proximal left anterior descending coronary artery, and proximal right coronary artery, and were denied as the Z scores (13). Z score = the internal dimension of the coronary artery expressed as the number of SD units normalized for body surface area of 2.0 or more. This study was approved by the Ethics Committee of Children's Hospital of WUXI, and the informed consent forms were obtained from the parents of all subjects.

All patients are divided into three groups: (1) healthy control group (HC); (2) patients with coronary artery lesion (CAL); (3) patients with non-coronary artery lesion (NCAL).

### Flow Cytometry Measured ROS Production (14)

Venous blood samples using EDTA as an anticoagulant were collected from KD patients before intravenous immunoglobulin and oral aspirin administration on the day of admission, sent for examination within 2 h. Flow cytometry assay using dihydrorhodamine (DHR) for the measurement of the neutrophil respiratory burst in whole blood. DHR is only weakly fluorescent and can be passively loaded into neutrophils. Upon neutrophils activation, ROS produced by NOX2 is transformed by superoxide dismutase (and possibly myeloperoxidase) into hydrogen peroxide (H_2_O_2_) that oxidizes DHR into the strongly fluorescent molecule, rhodamine (RHO).

Transfer 50 μl whole blood sample into each of two polypropylene tubes labeled “Basal” and “PMA-stimulated.” Add 50 μl PBS (phosphate buffered saline) or 10 μg/ml PMA 50 μl (phobol myristate acetate, Fluka USA) to appropriate tubes and incubate for 15 min at 37°C in shaking water bath. Twentyfive microlitre DHR were added to each tube. After mixing, 37°C water bath was taken for 5 min; add 1 ml hemolysin to each tube; after 10 min, 1,500 rpm centrifugation for 5 min. Abandon the supernatant, add 4 mL PBS, wash twice, 1,000 rpm centrifugation for 5 min. Abandon the supernatant, add 500 μl PBS for detected.

To determine neutrophil oxidative burst capacity, DHR conversion into the fluorophore rhodamine (RHO) was detected by flow cytometry fluorescence at 488 nm from detectors both below (bottom read) or above (top read) the samples. After neutrophils door setting, they provide basic graphics to show the basic level of neutrophils activation. On the flow cytometry, the number of DHR positive cells was counted after granulocyte gating. The unstimulated tube provides a basic figure to show the basic level of granulocyte activation. Under normal conditions, the DHR stained positive cells in the tube stimulated by phorbol ester (PMA) should be significantly increased. After PMA stimulation, determine whether the neutrophil activation state response has related functional defects.

### Plasma Levels of BNP

Blood samples were placed in EDTA-treated tubes, transported to the laboratory department of our hospital, and analyzed by fluorescence immunoassay (Ortho Clinical Diagnostic, Johnson & Johnson, USA). The measurable range of the BNP assay was 125–100,000 ng/L.

### Statistical Analysis

Statistical analyses were performed using SPSS version 20.0 for windows (SPSS Inc., Chicago, IL, USA). The measurement data of each group were expressed as mean ± SD, and the independent sample *T*-test was used for comparing the basic level of neutrophils activation with PBS and the level of BNP. Intergroup differences neutrophils activation with PMA were analyzed with F-test, *P* < 0.01 was used for statistical significance. ROC curve analysis was performed to determine the cut-off values of neutrophils activation with PBS. Binary logistic regression analysis was used to statistically analyze risk factors.

## Results

### Children Characteristics

A total of 58 children were recruited from January to December in 2019 before intravenous immunoglobulin therapy. Twenty patients belong to the normal control group (HC), including 12 males and 8 females. Twenty patients with coronary artery lesion (CAL) include 11 males and 9 females; 38 patients with non-coronary artery lesion (NCAL) include 14 males and 4 females. The levels of WBC and ESR in KD group was higher than that in HC group, which was consistent with the characteristics of KD. In addition, there are no significant differences in gender, age, and weight between these groups (Table 1).

**Table 1:** Comparison of laboratory data and clinical characteristics between KD groups and the control group.

	**HC**	**KD (CAL)**	**KD (NCAL)**
*N*	20	20	38
Age (months)	21.50 ± 11.40	25.55 ± 22.77	20.75 ± 19.76
Male, *n*	8	26	12
Weigh (kg)	13.84 ± 6.36	12.09 ± 3.89	13.38 ± 5.73
WBC (× 10^9^)	6.12 ± 1.28	16.63 ± 34.91	13.70 ± 5.34
ESA (mm/hr)	16.55 ± 4.81	53.23 ± 19.54	38.38 ± 15.57

### Differences of the Activation Ratio of Neutrophil in KD Among CAL, NCAL, and HC Groups

**Figure 1:**
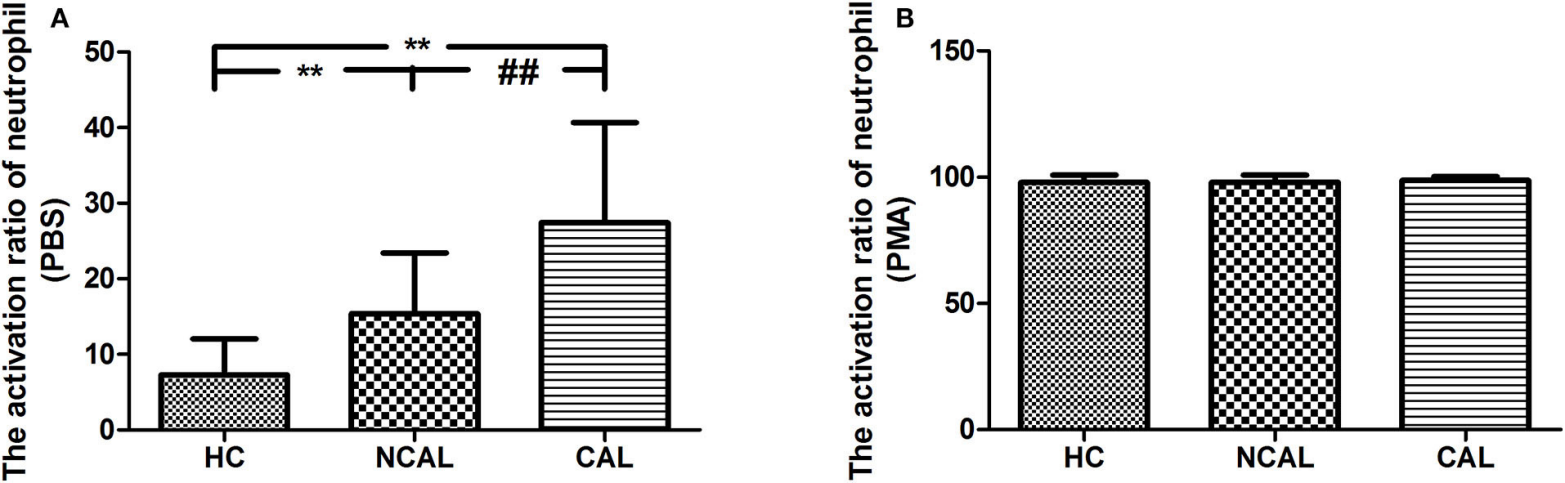
The basal activation ratio of neutrophil with PBS **(A)** and PMA **(B)** in KD with CAL group, NCAL group, and HC group. Data are expressed as means ± SD. **p < 0.01 vs. HC group, ^*##*^*p* < 0.01 vs. NCAL group.

To confirm the activation of neutrophil, we detected the activation ratio of neutrophil in three groups of CAL, NCAL, and HC reflecting ROS production. As shown in Figure 1, the basal neutrophil activation ratio with PBS in KD with CAL group was significantly higher than that in NCAL group (*t* = 4.79, *P* < 0.01). Besides, an obvious difference was found between KD groups (NCAL or CAL) and HC group (*t* = 6.424, *P* < 0.01, *t* = 4.79, *P* < 0.01, respectively). Furthermore, there was no significant difference of activation index between the three groups after PMA stimulation (*F* = 0.476, *P* = 0.478).

### Differences of BNP Level in KD Among CAL, NCAL, and HC Groups

**Figure 2:**
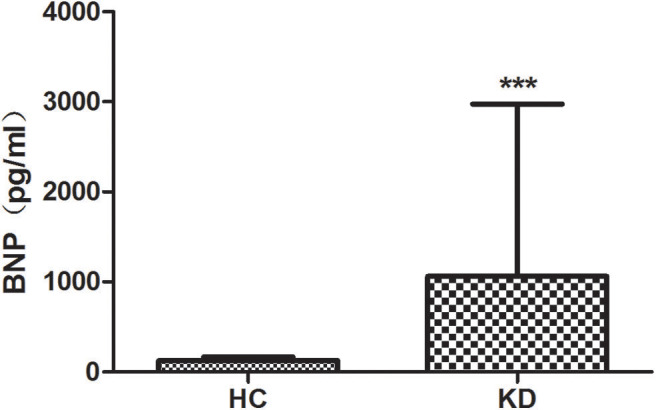
The plasma lever of BNP in KD with CAL group, NCAL group, and HC group. Data are expressed as means ± SD. ***p < 0.001 vs. HC group.

We examined the level of plasma BNP in all groups (Figure 2). Results showed that the plasma level of BNP is higher in KD group (1061.16 ± 1914.21) than that in HC group (125.35 ± 38.25) with significant difference (*P* < 0.01). However, the level of BNP between CAL (1064.78 ± 2064.01) and NCAL (1055.93 ± 1651.93) did not show significant difference (*t* = 0.16, *P* = 0.982).

### The ROC Curve of Neutrophil Activation Ratio of PBS

**Figure 3:**
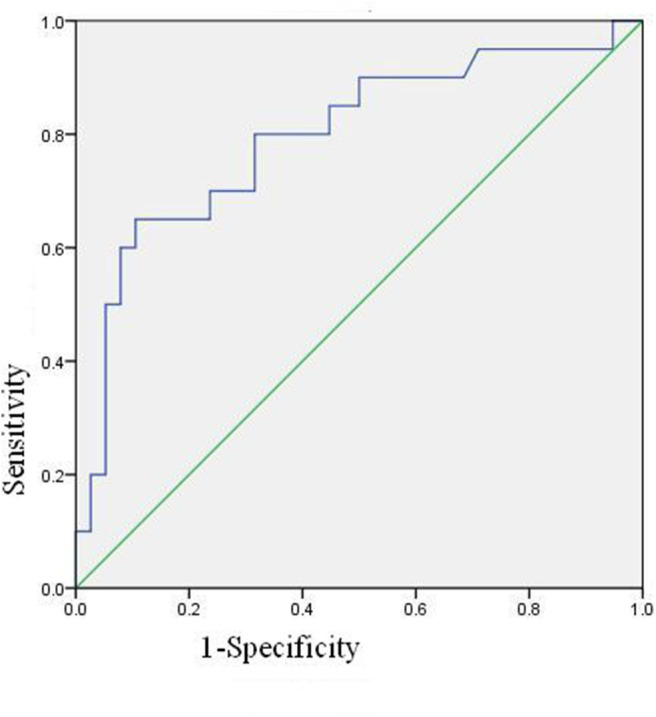
ROC curves describing the basal activation ratio of neutrophil with PBS.

The ROC curve of neutrophil activation ratio with PBS for predicting coronary artery damage is shown in Figure 3. The area under the curve is 0.795 (95% confidence interval is 0.000–0.925, *p* = 0.000), which means the ROC curve is credible and of statistical significance. When the cut-off value is 24.4, i.e., PBS is more than 24.4, the predicted results are positive, and the sensitivity is 13/20 = 65%, the specificity is 34/38 = 89.47%.

Binary logistic regression analysis suggested that basal activation ratio of neutrophil was the risk factor of coronary artery damage, and the risk of coronary artery damage increased by 1.123 times for every unit increased (*P* = 0.001, 95% confidence interval is 1.047–1.204).

## Discussion

In the present study, 20 healthy controls and 38 KD patients were recruited. It was found that the activation rate of neutrophils in KD group was significantly higher than that in HC group under PBS basal control, while that in KD group with CAL was higher than that in NCAL group. Risk analysis indicated that neutrophil activation was a high risk factor for coronary lesion in KD. The higher the basal activation rate of neutrophil, the more likely the occurrence of coronary lesion.

Endothelial cells (ECS) play an important role in the physiological and pathological processes of hemostasis, inflammation, and angiogenesis. The imbalance of ROS production and antioxidant defense system is the main cause of endothelial dysfunction, which leads to the damage of endothelial cells or vascular smooth muscle cells (15). Because of the damage of blood vascular walls, vessel dilation might occur (16). These pathological changes may be the cause of vasculitis and coronary artery lesion in Kawasaki disease.

Neutrophil respiratory burst producing ROS as a means of attacking pathogens is the prerequisite as an efficient first line, and it is an important mechanism through which macrophages protect the host (17, 18). However, the imbalance of production and the detoxification ability of biological system to reactive intermediates of ROS might cause oxidative stress. Oxidative stress contributes to the mechanism of inflammation and tissue injury. The ROS acts as both a signaling molecule and a mediator of inflammation (19). The most important mechanism for neutrophils phagocytic and antimicrobial activity is the release of granular products (i.e., metalloproteinase [MMP]-8 and -9, myeloperoxidase [MPO], neutrophil gelatinase-associated lipocalin release cytokines), leading to the damage of membranes; proteins and DNA are believed to play a critical role in vascular disease (20), bring about further macrophage recruitment and the proliferation of smooth muscle cells within the vascular wall. In addition, protease secretion leads to endothelial damage of the coronary vessels, exposing thrombogenic collagen, and predisposing the vessels to thrombus formation (21–23). To clarify the relationship between neutrophil respiratory burst and KD with CAL, a multi-parameter flow cytometry technique using dihydrorhodamine 123 (DHR) was applied. Results showed that the basal neutrophil activation ratio with PBS in KD with CAL group was significantly higher than that in the NCAL group, indicating that neutrophil respiratory burst plays an important role in the occurrence of CAL in KD. The ROC curve of neutrophil activation ratio with PBS is credible and of statistical significance. Binary logistic regression analysis suggested that basal activation ratio of neutrophil was the risk factor of coronary artery damage. Besides, the neutrophils can be activated by PMA in healthy adults and children, but not in some immune deficient diseases such as chronic granulomatous diseases. So after PMA stimulation, the neutrophil activation of three groups without functional defects have no significant difference.

BNP can stimulate macrophages to produce ROS and increase NO2 release by NADPH oxidase. It can also modulate cytokine production in several cell types, up-regulating the production of IL-10 by macrophages and inhibiting IL-12 and TNF-a release by dendritic cells (DCs), suggesting an anti-inflammatory cytokines profile induction (24). The anti-inflammatory effect of BNP may be also related to the inhibition of ROS formation by neutrophils (25). BNP inhibited the release of ROS in a cGMP/ PKG dependent manner (26). BNP may present both anti- and proinflammatory actions. These findings have a number of potential therapeutic sequelae. In our study, the plasma lever of BNP is significantly higher in KD group than that in HC group.

## Conclusion

In conclusion, our study showed that neutrophil respiratory burst evidenced by ROS production was significantly increased in the acute stage of KD patients, which was more pronounced in the CAL group and could be used as an indicator to predict CAL. BNP levels are elevated in the plasma of children with Kawasaki disease but there is no difference between KD with and without CALs.

Although we demonstrated that ROS and BNP are important to the pathogenesis of KD, the specific mechanisms still need further studies.When neutrophils were activated by microbial or inflammatory stimuli, the web-like structures known as neutrophil extracellular traps (NETs) were released. NETs are composed of chromatin DNA and neutrophil granule protein. Several of the molecules decorating the NETs (e.g., MPO, ds-DNA, histones, etc.) are autoantigens in systemic autoimmune diseases such as antineutrophil cytoplasmic antigen (ANCA)-positive vasculitis and systemic lupus erythematosus (SLE) (27). Next, we want to investigate the relationship between NET and Kawasaki disease.

## Data Availability Statement

The raw data supporting the conclusions of this article will be made available by the authors, without undue reservation.

## Ethics Statement

The studies involving human participants were reviewed and approved by Ethics Committee of Children's Hospital of WUXI (WXCH2015-10-003). Written informed consent to participate in this study was provided by the participants' legal guardian/next of kin.

## Author Contributions

JH, WQ, JL, and HL: conceptualization. ZY, TX, and QH: formal analysis. HL and JL: funding acquisition. WQ, TX, LJ, QH, and YW: methodology. JH and WQ: writing-original draft. JH, JL, and HL: writing-review and editing. All authors contributed to the article and approved the submitted version.

## Conflict of Interest

The authors declare that the research was conducted in the absence of any commercial or financial relationships that could be construed as a potential conflict of interest.
